# Real-time reconstruction and visualisation towards dynamic feedback control during time-resolved tomography experiments at TOMCAT

**DOI:** 10.1038/s41598-019-54647-4

**Published:** 2019-12-05

**Authors:** Jan-Willem Buurlage, Federica Marone, Daniël M. Pelt, Willem Jan Palenstijn, Marco Stampanoni, K. Joost Batenburg, Christian M. Schlepütz

**Affiliations:** 1 Centrum Wiskunde & Informatica, Amsterdam, The Netherlands; 2 Swiss Light Source, Paul Scherrer Institute, Villigen, Switzerland; 3 Institute for Biomedical Engineering, ETH Zürich, Zürich, Switzerland; 4 Mathematical Institute, Leiden University, Leiden, The Netherlands

**Keywords:** Characterization and analytical techniques, Imaging techniques

## Abstract

Tomographic X-ray microscopy beamlines at synchrotron light sources worldwide have pushed the achievable time-resolution for dynamic 3-dimensional structural investigations down to a fraction of a second, allowing the study of quickly evolving systems. The large data rates involved impose heavy demands on computational resources, making it difficult to readily process and interrogate the resulting volumes. The data acquisition is thus performed essentially blindly. Such a sequential process makes it hard to notice problems with the measurement protocol or sample conditions, potentially rendering the acquired data unusable, and it keeps the user from optimizing the experimental parameters of the imaging task at hand. We present an efficient approach to address this issue based on the real-time reconstruction, visualisation and on-the-fly analysis of a small number of arbitrarily oriented slices. This solution, requiring only a single additional computing workstation, has been implemented at the TOMCAT beamline of the Swiss Light Source. The system is able to process multiple sets of slices per second, thus pushing the reconstruction throughput on the same level as the data acquisition. This enables the monitoring of dynamic processes as they occur and represents the next crucial step towards adaptive feedback control of time-resolved *in situ* tomographic experiments.

## Introduction

Synchrotron tomography beamlines are powerful tools for obtaining high-resolution interior visualisations of a wide variety of opaque specimens with applications in life sciences, energy research, new materials, and many other fields. Thanks to advances in CMOS detector technology during the last decade and to the high photon flux available at state-of-the-art tomographic microscopy endstations, it is now possible to acquire the raw data required for computing a full 3D snapshot in well under one second at micron resolution, promoting the use of tomographic microscopy for time-resolved 3D imaging of interior dynamics^1–3^. For example, the GigaFRoST detector^4^ in use at the fast tomography endstation of the TOMCAT beamline at the Swiss Light Source (PSI) can acquire up to 1255 full frame projection images of size 2016 × 2016 pixels, each second, and directly stream them to a data backend that is capable of receiving and storing this 7.7 GB per second in a ring buffer. Efficient handling of these large data rates associated with time resolved tomographic experiments is a major challenge: large bandwidths for data transfer and data storage are required as well as sufficient computational resources for performing tomographic reconstruction and subsequent analysis. Even with modern efficient software packages and high-performance computational resources, the rate at which the data can be processed and analysed is often several orders of magnitude slower than these high rates of data acquisition. At most beamlines, typically the tomographic reconstruction of a high resolution volume takes at least a few minutes, with differences related to the used algorithm and available computational resources (e.g.^5–8^).

Direct visual feedback during a time-resolved experiment is of key importance for streamlining the efficiency of the physical imaging setup and the computational pipeline, which jointly determine the overall utilisation of the synchrotron beamline. At TOMCAT the beamline operator currently makes use of two types of direct visual feedback: (i) by observing the raw projection images it is possible to locate regions of interest in the sample, as long as these regions can be clearly identified in the projections, which is not always the case; (ii) by reconstructing a single axial slice on-the-fly and observing it during the experiment it is possible to get an initial grasp of the internal structure of the sample^7^. This limited form of real-time feedback during the experiment does not provide detailed insights in the 3D structure of the sample, particularly important for strongly anisotropic objects (e.g. fibres), where virtual tomographic slices with different orientations can look very different and provide valuable complementary information.

The lack of real-time 3D feedback represents a major obstacle to the efficiency of in particular dynamic imaging experiments. On one side rapid access to tomographic volumes could increase the success chances of the measurement campaign as it permits fast reaction towards the optimisation of the beamline parameters and data collection protocols to guarantee sufficient image quality to subsequently extract the relevant physical information. Acquisition problems that result in imaging artefacts, such as detector misalignment, could be resolved on-the-fly, thereby making much more effective use of expensive and scarcely available synchrotron beamtime. On the other side, *in situ* experiments often require event-driven imaging, where the timing of the operations performed on the sample (e.g. heating, wetting) and the timing of the image acquisition are tightly connected. Examples include stress loading of construction materials, water uptake of textiles, and migration processes inside batteries. By observing the interior dynamics in real-time during the experiment, the control parameters could be adjusted on-the-fly in response to the observed phenomena. Finally, real-time feedback on the 3D structure of the sample would provide the ability to match the number of acquired tomographic volumes to the observed dynamics leading to a potentially substantial reduction of the total amount of produced data, not irrelevant during time-resolved experiments with kHz frame rate detectors, and to a maximisation of the information content in the stored datasets.

Because of the importance of direct 3D feedback during the experiment, previous research has focused on reducing the required computation time for obtaining a 3D snapshot of the scanned object, often through computational advances. One approach is to use supercomputing facilities to massively parallelise the various computations^9,10^, significantly reducing the required computation time. For example, by using 32 K supercomputing nodes, it is possible to compute full iterative 3D reconstructions in minutes^9^. However, supercomputing facilities typically have to be shared with other users, and computing time may not be available at the time it is needed during the experiment. A different approach is to use smaller clusters of GPU-equipped machines in combination with advanced software packages that can efficiently stream data to and from the GPUs^11^. As an example, with this approach it is possible to compute 3D snapshots of moderately sized problems in several seconds using six GPUs^11^. Despite these advances, real-time (i.e. sub-second) reconstruction and 3D visualisation during time-resolved tomography experiments is still out of reach.

In this article we present a data processing pipeline for real-time reconstruction and visualisation during the imaging experiment. Our main contribution is that we combine recent improvements in ultra-fast detector technology, networking, and tomographic reconstruction. This is a complex engineering effort, which requires combining expertise from multiple disciplines. Although several groups have shown the potential of real-time reconstruction at synchrotron light sources, we demonstrate for the first time a fully implemented pipeline for real-time reconstruction and visualization of time-resolved tomographic experiments. Instead of computing an entire 3D snapshot of the scanned object, our approach computes multiple arbitrarily oriented slices. The pipeline is based on combining the GigaFRoST detector system^4^, which provides direct access to newly acquired projections, with the recently published RECAST3D software^12^, which enables real-time visualisation of arbitrary oriented slices by directly reconstructing the slices from the measured projections. The image reconstruction part of our pipeline runs on a single GPU-equipped workstation, thereby providing an imaging solution that can be implemented at the beamline in a straightforward manner without need for on-demand access to compute and network resources at a supercomputing facility. By setting up three orthogonal slices across the three main axes of the imaging system, a quasi-3D visualisation of the interior structure of the sample is obtained. During the experiment, the visualisations are automatically updated in real-time, ensuring that the most recent state of the scanned object is always shown. Since the visualised slices can be re-positioned and tilted in arbitrary directions at any time, the visualisation can be dynamically aligned with features of interest of the scanned object providing key information to the scientists in real time, unlocking the possibility to take further action towards the optimisation and control of the imaging and experimental parameters.

## Method

To achieve real-time visualisation of tomographic experiments, our pipeline includes two main parts: a detector component that provides direct access to acquired projections in real time, and a software component that can process the acquired data and visualise results in real time. In the realisation we present here, the detector component is implemented using the GigaFRoST detection and readout system^4^, while the software component consists of the RECAST3D real-time reconstruction and visualisation software and streaming architecture^12^. We will first discuss in more detail the elements of both components relevant to the presented pipeline, and then explain how the two components were integrated.

### GigaFRoST

The GigaFRoST^4^ is a detection and readout system that can acquire and stream data continuously at 7.7 GB/s to a dedicated backend server. Coupled to a scintillator screen and efficient optics, this hardware unlocks unprecedented time-resolved tomographic microscopy capabilities, including simultaneously an elevated time resolution and the ability to follow dynamic phenomena for a long time. Built on top of a commercial CMOS sensor, it does not have an on-board RAM as is typically the case for high frame rate cameras on the market optimised for burst operation, but it directly streams the acquired data through eight fibre-optics connections to a backend server. In this way the number of images that can be acquired in one sequence is not limited by the internal detector memory and sustained fast data acquisition is possible. The backend server collects the data blocks dispatched by the detector and reassembles them into projection images in a ring buffer. These frames can then be sent to any downstream process (e.g. reconstruction pipeline and file writer). For this purpose, a publishing process posts the data using a distributed message passing protocol based on ZeroMQ streams. In this way, simple direct access to the acquired images is guaranteed: any downstream process can subscribe to the ZeroMQ data stream published by the backend.

### RECAST3D

**Figure 1:**
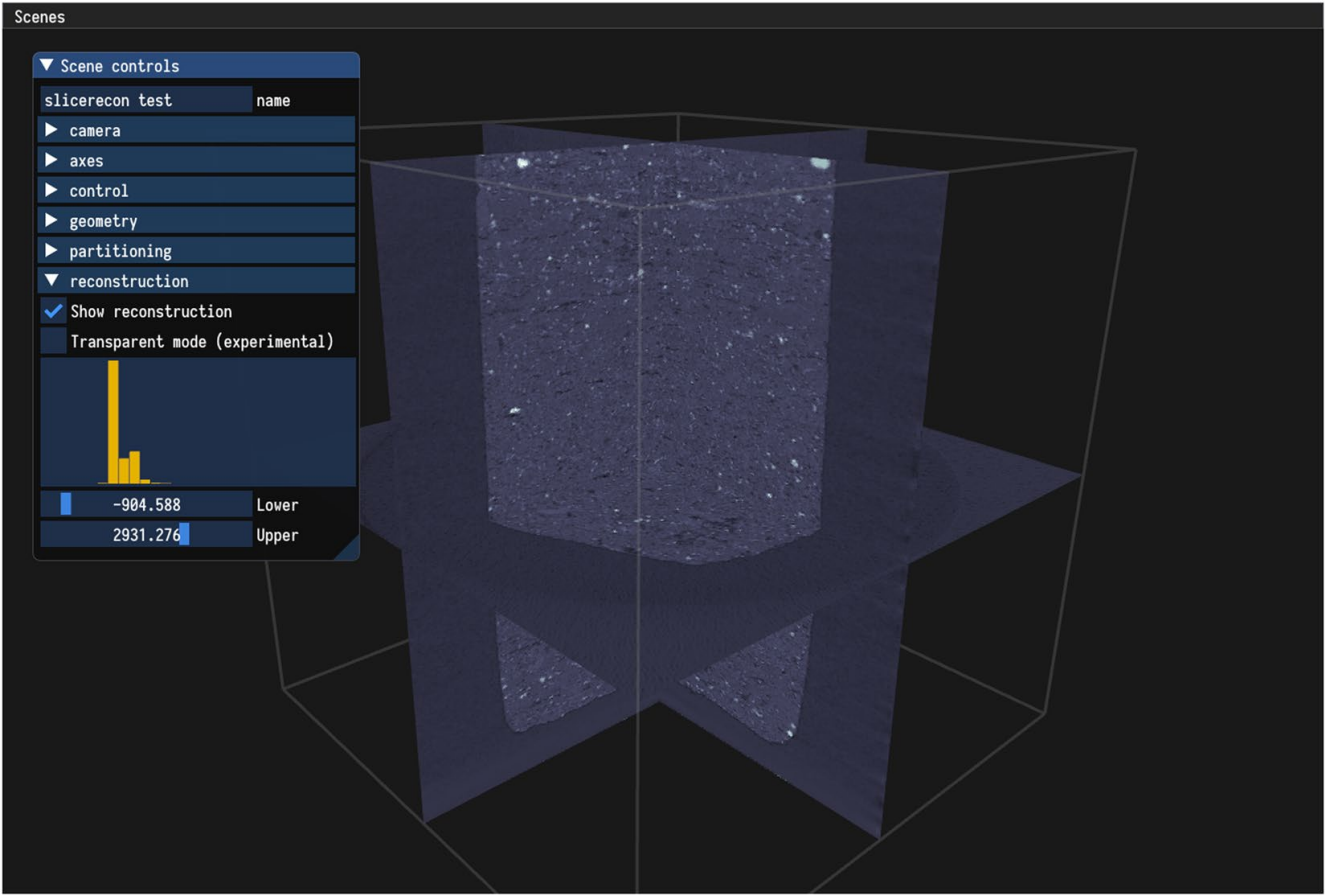
Overview of the RECAST3D interface. A number of arbitrarily oriented slices are chosen by a user using a simple, intuitive interface. Reconstructions are continuously updated as new data comes in, giving real-time visual feedback during time-resolved tomography experiments. The slices can be reoriented as necessary without any noticeable impact on the reconstruction time. Various controls for adjusting visualisation and reconstruction parameters are shown on the left.

The RECAST3D framework^12^ provides a quasi 3D reconstruction of the scanned object by simultaneously reconstructing and visualising a set of arbitrarily oriented tomographic slices, which can be dynamically chosen by the user and are constantly updated in real time (Fig. 1). To derive a computationally efficient technique for reconstructing such arbitrarily oriented slices, we first note that the reconstruction problem in tomography can be modelled as a linear system $A{\bf{x}}={\bf{b}}$. Here, **x** has a component for each of the ${N}_{x}\times {N}_{y}\times {N}_{z}$ voxels in the discretised representation of the object being imaged, **b** is the collection of (preprocessed) intensity measurements obtained on the detector, and *A* is the forward-projection operator, with *a*_*ij*_ the contribution of voxel *j* on intensity measurement *i*. *A* is sparse with only $O({N}_{\phi })$ nonzero entries in each column, where ${N}_{\phi }$ is the number of projection angles. This sparsity can be used to efficiently compute reconstructed slices using the filtered backprojection (FBP) technique. FBP is a popular reconstruction technique, because it is computationally efficient, straightforward to implement^13^, and provides high-quality reconstructions if a sufficient number of projections is available and the noise level is limited. An FBP reconstruction consists of two steps: first, the data is filtered, and afterwards, the filtered data is backprojected into the image array to produce the final reconstruction. Using the notation above, FBP can be written as

1 $${\bf{x}}={A}^{T}C{\bf{b}},$$

where *C* is a filtering operation that performs 1D convolutions on each individual row of the projection images.

The key to the RECAST3D approach is that only a limited number of components of **x** needs to be computed for arbitrarily oriented slices, namely those corresponding to voxels of the slices. Without loss of accuracy, this can be done efficiently using an FBP algorithm. First, filtered projections ${\bf{y}}=C{\bf{b}}$ can be computed relatively easily in real-time, because the computation is trivially parallel (each row of each projection can be filtered independently) and because the 1D convolution operations can be efficiently calculated as element-wise multiplications in the Fourier domain. Second, since each column of *A* only contains ${N}_{\phi }$ nonzeros, the reconstructed value for a single voxel at any arbitrary position in the volume is given by a weighted sum of ${N}_{\phi }$ (filtered) data elements (see Eq. (1)). As a result, the reconstruction of an arbitrarily oriented slice with *n*^2^ voxels requires only $O({n}^{2}{N}_{\phi })$ operations, which is significantly less computationally demanding than the reconstruction of the full 3D *n*^3^ voxels volume ($O({n}^{3}{N}_{\phi })$ operations), since *n* is typically as high as a few thousand. In addition, for the reconstruction of an arbitrarily oriented slice, the system in Eq. (1) can be reduced to include only the information relevant to the voxels of interest:

2 $$[\begin{array}{c}{{\bf{x}}}_{{\rm{slice}}}\\ {{\bf{x}}}_{{\rm{other}}}\end{array}]={[{A}_{{\rm{slice}}}{A}_{{\rm{other}}}]}^{T}{\bf{y}}\,\Rightarrow \,{{\bf{x}}}_{{\rm{slice}}}={A}_{{\rm{slice}}}^{T}{\bf{y}}.$$

Because this reduced system still represents a backprojection operation, existing efficient and highly flexible GPU based backprojection routines (e.g. those found in the ASTRA toolbox^14^) can be readily used to compute arbitrarily oriented slices without modification. The local properties exploited in the presented approach are specific to the FBP algorithm. Other reconstruction techniques, such as *gridrec*^15^, which revolve around regridding of the data in Fourier space, can be up to 20 times faster for full 3D data sets than FBP^16^, but cannot be restricted to reconstruct arbitrarily oriented slices, since they rely on a Fourier inversion of the entire volume.

The quasi-3D reconstruction pipeline of RECAST3D^12^ is built upon a message-passing protocol between a visualisation tool for reconstructed slices and a reconstruction server. The reconstruction server holds (preprocessed) tomographic projections in memory, and is able to reconstruct arbitrarily oriented slices from this data on demand, e.g. by dynamic selection by the user in the visualisation interface (Fig. 1). A low-resolution 3D preview is provided by the reconstruction server as well, to aid the user while selecting slice positions and orientations. The active set of projections is continuously being updated during the scan, ensuring that the current state of the scanned object is always visualised.

### Integration

The GigaFRoST system and the RECAST3D reconstruction pipeline are linked through a distributed message passing protocol based on ZeroMQ streams, which abstract away much of the network communication. The reconstruction server subscribes to the backend server stream to obtain, in real time, the projections from the tomographic measurement. Currently, a single workstation is used for reconstruction and visualisation. This workstation consists of an NVIDIA Quadro K6000 GPU with 12GB on-card memory, and two Intel Xeon CPU E5-2680 v2 CPUs. Projections are received from the backend server over a 10 Gbit network connection.

**Figure 2:**
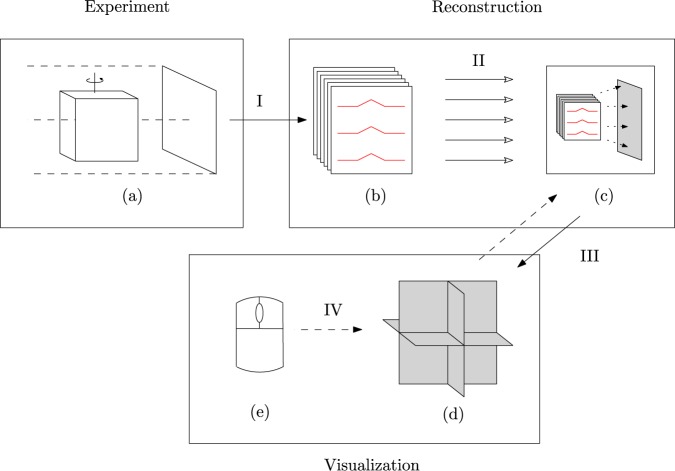
A tomographic measurement (**a**) leads (I) to a stack of projection images (**b**). The rows of these images have to be filtered (in red), which can be done in parallel (II). The filtered projection images can be used to reconstruct individual slices (in grey), by local backprojection operations (**c**). These slices can be shown together (**d**). The visualisation software can request reconstructions (III), in particular upon interactive slice rotation and translation (IV) by the user (**e**).

**Figure 3:**
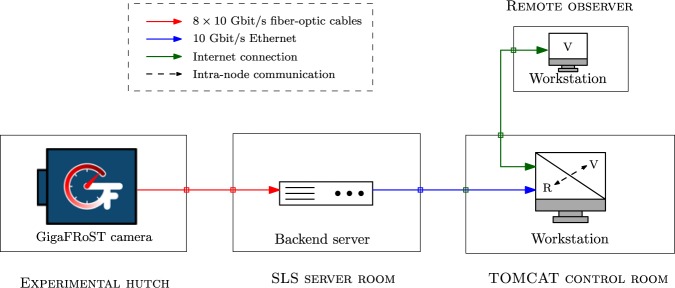
The IT infrastructure used in the real-time reconstruction pipeline. The data from the detector is received by a ring buffer on the backend server. This data is then streamed to the reconstruction software (R) currently running on a single workstation in the control room. The communication between the visualisation software (V) and the reconstruction software now happens within this single workstation.

The tomographic measurement consists of multiple scans. In each scan, a data frame of ${N}_{\phi }$ projections is recorded. These projections are preprocessed and filtered as they come in by the combined 40 independent hardware threads of the CPUs, and then uploaded to GPU memory. The implementation also supports optional phase retrieval using the Paganin method^17^. The GPU holds two buffers, each large enough to store a data frame. The active buffer is always the latest complete data frame that has been fully processed and uploaded. New slice reconstructions are triggered in two ways: (i) when the user interactively chooses a new slice to be visualised, typically by translating or rotating one of the active slices in the visualisation tool, and (ii) when a new data frame has been fully processed and uploaded. A reconstruction is realised by a single backprojection operation onto a slice from the data in the active buffer, cf. Eq. (2). Additionally, a low-resolution 3D volume is reconstructed when a new data frame has been fully processed and uploaded. A separate process handles the connection to a visualisation server, sending new reconstruction data when it becomes available. Optionally, remote observers can connect through an internet connection to the reconstruction server, and can request slice reconstructions independently from the on-site user. The overall setup is illustrated in Fig. 2. The IT infrastructure is illustrated in Fig. 3.

Benchmarking results of the current implementation are presented in Table 1. There are two main performance aspects to consider. The first aspect is the time it takes to process and upload a data frame to the GPU. From the results, we see that the setup is capable of processing a set of 400 projections with 768 × 520 pixels and uploading it to the GPU well within a single second. One reason we do not use the full detector resolution, is to ensure that the projection data fits in the memory of the used GPU. The GPU memory usage is dominated by the active and passive projection buffers. Using the full GigaFRoST detector resolution of 2016 × 2016 for 400 projections would lead to a memory usage for the projection buffers of roughly ${2016}^{2}\times 400\times 2\times 4\,{\rm{B}}\approx 13\,{\rm{GB}}$ when the values are stored in single precision. The other buffers, one to store a single reconstructed slice and one for the low-resolution 3D volume, take up a negligible amount of memory. Besides affecting memory usage, limiting the size of the projections also reduces the computational load in the preprocessing step, as well as the bandwidth required to upload the data to the GPU in time. The restriction on the size of the projection data can be lifted by using a GPU with more memory, or, as we discuss later, by moving to an implementation that uses multiple GPUs. We are currently able to realise a raw data bandwidth of roughly 4Gbit/s. If required, only a part of the data is selected for use in the real-time reconstruction, to ensure that the incoming data can be processed and uploaded in time. In practice, this means that the reconstruction shown in the visualiser always comes from data that has been recorded less than one second earlier. The second performance aspect to consider is the time it takes to reconstruct an arbitrary slice from the active data buffer. Because of the way slices are reconstructed, it is convenient to choose a fixed size for the slices regardless of orientation. This is a parameter that can be set by the user. We use voxel-driven backprojection, and sampling is done by interpolating values of the projection images. For this benchmark, we choose to reconstruct slices with a relatively high resolution of 1024 × 1024 pixels to obtain a conservative estimate for the maximum reconstruction time. The total response time between the reconstruction and visualisation server, i.e. the time between requesting a slice reconstruction and receiving it, is less than 100 ms, realising the goal of being able to examine the imaged sample in real time. In summary, we see that with this implementation the time elapsing between the selection of a new slice by the user and its visualisation is negligible, so that it is as though fully reconstructed 3D data is available.

**Table 1:** Benchmark results for the reconstruction pipeline.

Process	Upload	Preview	Slice	Total For Three Slices
386.3 ms	197.9 ms	49.1 ms	31.5 ms	727.9 ms

Each data frame contains 400 projections with 768 × 520 pixels. The reconstructed slices consist of 1024 × 1024 pixels. The reconstructed 3D preview consists of 128 × 128 × 128 voxels. Here, Process is the processing time for a single data frame, e.g. flat fielding and filtering. The total time to upload a data frame to the GPU is shown as Upload. The reconstruction time for processed data stored on the GPU for a 2D slice and a 3D preview is given as Slice and Preview respectively. Although many of the steps happen in parallel, a worst-case estimate for the processing of a single data frame and reconstruction of three arbitrary slices can be found by the sequential time computed as Process + Upload + Preview + 3 × Slice. This is estimate is shown as Total For Three Slices.

The connection between the backend server and the RECAST3D service is realised using a publish/subscribe pattern. The subscriber listens to messages sent by the publisher. In our case, a single message corresponds to one projection image. Using the ZeroMQ implementation of this pattern, message order is maintained between the publisher and subscriber, and messages are received at most once. However, there are no other strict guarantees on the messages. For example, it is not guaranteed that all messages are received by the subscriber. Our setup is mostly robust to missing messages, as the corresponding part of the buffer will be filled with zeros. When a backprojection operation using this buffer is executed, the missed projection images are then effectively ignored. In the worst case, this can result in missing angle artefacts when the number of dropped images is large, and it can reduce the overall intensity of the reconstructed image.

The employed scheme gives a lot of flexibility to the system. Listeners can subscribe and unsubscribe on demand, without requiring any additional logic to be implemented on the backend server. In the current implementation, messages are queued when the subscriber is overworked, and once this queue is full messages will start to drop. This can happen when we deal with particularly high-throughput data. One possibility to resolve this issue is to only send every *N*th data frame to RECAST3D, for some appropriate value of *N*, while all get saved to disk, which would require inserting a ZeroMQ stream splitter into the stream.

In order to support higher resolution data sets, or to increase the number of data frames the pipeline can process, we have to move beyond using a single GPU. While currently only a single workstation is used for reconstruction and visualisation, the framework is scalable. Multiple compute nodes can be used for processing and reconstructing in parallel. One way to achieve this is to split a data frame into groups of projections and distribute them over a number of GPUs. Each group of projections can be filtered independently. When a slice reconstruction is requested, each GPU performs a backprojection with its local group of projections leading to a contribution to the reconstructed slice. Next, we perform a single distributed step over all GPUs, where the contributions are summed to obtain the slice reconstruction for the full projection set. We note that this summation is performed only on 2D data, limiting the required communication between GPUs as well as the computational cost. This parallelization method is possible because the backprojection operator is linear. In the current implementation, the CPU-based pre-processing could form a bottleneck to the scalability. However, expensive steps such as filtering the projections could be offloaded to a GPU. Based on the results obtained with a modest workstation, we expect that when a small-size cluster of about 8 GPU nodes is used, full-resolution tomographic reconstructions with a finer temporal resolution should be achievable.

It is also possible to further optimise the GigaFRoST system for the specific application of real-time visualisation and feedback. The service running on the GigaFRoST backend server is currently implemented as a ring buffer, and it does not guarantee streaming out the frame data in consecutive order. This is in order to optimise performance when it is under heavy load. Although ZeroMQ streams guarantee maintaining message order, this means we cannot rely on this in practice, because images from successive data frames can intermix and throw off the processing and updating of the active buffer. To circumvent this issue for the present experiments, we chose a sufficiently long wait period between individual scans. After a planned modification to the service running on the backend server, this should no longer be necessary in the future.

Our primary aim of the proposed pipeline is not to outperform the already established pipeline running on a large CPU cluster in the reconstruction of complete 3D data sets. The main advantages of the proposed pipeline over the existing pipeline are: (i) Slices with arbitrary orientations though the volume can be reconstructed. This would not be possible on the existing production cluster, which relies on the gridrec algorithm instead of FBP and would thus first need to reconstruct the full volume before being able to compute and visualise an arbitrarily oriented data slice through the volume. Due to this, the performance gain for visualising arbitrarily oriented slices is over a factor of 10 compared to the production pipeline. (ii) The current production environment lacks the interactive visualisation environment provided by RECAST3D, and thus also the capability to choose and adjust the requested slice positions dynamically during the running measurement. (iii) The proposed system is designed to run on a very simple and modest compute infrastructure compared to the relatively large CPU cluster required by the existing pipeline.

## Scientific Applications

In this section, the new features, current benefits and future potential of the presented real-time reconstruction and visualisation tools are illustrated on a selected case study which is, however, representative of a wide range of dynamic phenomena.

Fluid uptake characteristics and transport mechanisms in fibrous materials are widely and intensively studied on a very fundamental level, both experimentally^18,19^ and through models and simulations^20,21^, for a variety of technical applications, ranging from the impregnation of carbon fibre composite materials with a fluid polymer matrix, via the wettability and absorption of ink in paper and cloth-based carrier materials during ink jet printing, to the functionalisation of wearable textiles to control their water-repellent or moisture absorbing and transporting properties.

The wicking behaviour of a single yarn thread is investigated in a simple dynamic model experiment. A yarn is essentially a spun bundle of individual fibres. Depending on the fibre material, size distribution and homogeneity, and the tension and twist applied during spinning, the volume, distribution, shape, and inter-connectivity of pore spaces within the yarn differ significantly and, in turn, crucially affect the water transport and distribution within the yarn.

**Figure 4:**
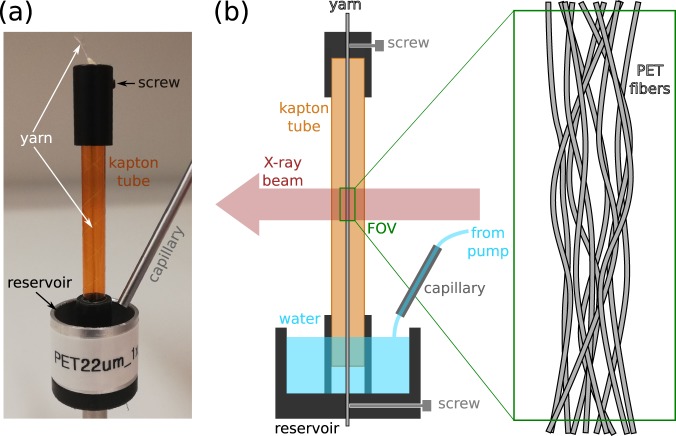
Experimental setup. (**a**) Photo of the yarn sample holder. (**b**) Schematic drawing of the sample holder and measurement geometry.

Figure 4 shows a sketch and photo of the setup used in the experiment. A yarn has been fabricated from 96 polyethylene terephthalate (PET) fibres of 22 *μ*m diameter. It is mounted inside a vertically positioned kapton tube of ca. 6 mm diameter and 50 mm length and is subject to a slight amount of twist and tension. The bottom part of the kapton tube features an aperture to allow liquid to enter in order to get the lower end of the yarn in contact with water. The tube is placed into a larger reservoir holder into which one can inject the liquid from a remotely controlled syringe pump. The whole assembly is mounted on the rotation stage in the beamline hutch and can be positioned such that the yarn is centred along the rotation axis.

Edge-enhanced X-ray absorption images are produced using the filtered (20 mm pyrolitic graphite + 75 um W) white beam of a 2.9 T superbending magnet, converted to visible light with a 150 *μ*m thick LuAG:Ce scintillator (Crytur, Czech Republic), and recorded using the GigaFRoST camera coupled to a high numerical aperture microscope^22^ (Optique Peter, France) featuring an optical magnification of 4x. This results in an effective pixel size of 2.75 *μ*m. The scintillator was placed 320 mm downstream from the sample to obtain some degree of edge-enhancement from the weakly absorbing PET fibres. Projection images were cropped to a size of 384 pixels horizontally by 800 pixels vertically to capture the full extent of the yarn illuminated by the approximately 2.2 mm high X-ray beam.

The experimental challenge for this system is twofold: Firstly, more than one transport mechanism governs the evolution of the water content in the yarn. These processes inherently proceed at different speeds and can result in abrupt changes of the uptake velocity over time. To capture the fast dynamics, scan times for individual volume reconstructions need to be kept as short as possible, ideally of the order of 0.1–0.5 seconds. Secondly, the arrival time point of the liquid front at the measurement position, which lies 10–20 mm above the water surface level in the reservoir, is very unpredictable and varies considerably from specimen to specimen. Hence, the data acquisition needs to be sustained at high speeds over a long period of time, thus putting stringent demands on the data streaming and storage infrastructure. In the end, the interesting dynamics will be restricted to only a short period during this extended time series, rendering most of the data unimportant.

The experiment then proceeds as follows: First, the rotation of the dry sample is started and the acquisition of projection images with the GigaFRoST camera^4^ is initiated. To ensure an identical sample orientation for successive volume scans and to throttle the scan rate, we employ the so-called sequence mode for data acquisition^23^, where the collection of a series of 400 images over a 180 degree range is triggered by the position-sensitive output signal from the rotation stage every 720 degrees during continuous rotation. With the chosen exposure time of 1 ms for each projection, this results in a scan time of 0.4 seconds per scan and a scan period of 1.6 seconds.

We will now discuss three specific examples of capabilities that our approach enables in practice.

### Capability I: real-time alignment of the setup

One of the first steps in any tomographic X-ray imaging experiment is the assessment and optimisation of the reconstruction image quality. Usually this is incrementally adjusted through a series of alignment procedures and test scans which have to be reconstructed and examined individually after each alignment step. Parameters to be aligned and optimised may include the tilt and position of the rotation axis with respect to the camera or the propagation distance between the sample and the scintillator to achieve the right amount of edge-enhancement. Performing these alignment and optimisation steps is greatly simplified and accelerated by the availability of a live view of reconstructed slices. These steps are demonstrated in Movie S1 in the Supplementary Materials. The rotation axis is initially offset with respect to the centre of the camera by a few tens of pixels, resulting in the characteristic C-shaped artefacts of the individual fibres comprising the sample structure in the axial slices of the live reconstruction. By simply tweaking the rotation axis’ or camera’s position transverse to the beam direction with the corresponding translation stage, one can progressively improve the quality of the reconstructed sample structure until an adequate alignment has been achieved. Note that when measuring radiation-sensitive samples, the alignment step should naturally be performed with a dedicated alignment tool before mounting the real samples. The precise centring of the rotation axis is, however, not strictly necessary to conduct an experiment, as the actual location can be determined in the reconstruction process and a slight misalignment can be easily corrected *a posteriori* to improve the reconstructed image quality. Similarly, in cases where a precise alignment during the experiment may not be possible due to mechanical constraints, a non-centred axis position could be specified as an input for the real-time reconstruction via RECAST3D.

### Capability II: real-time sample positioning

While the above mentioned optimisation and alignment steps usually only need to be performed at the beginning of an experiment series, each sample to be measured has to be positioned correctly with respect to the rotation axis to ensure that the proper region of interest (ROI) is imaged. For many samples, like the yarns in this experiment, this is easily achieved simply by looking at the projection images. However, particularly when looking at smaller regions inside an extended sample, navigating to the correct ROI simply based on the radiographic projections is often not straightforward. Again, a live view of a small number of reconstructed slices through the volume can easily guide the navigation and ensure that the proper region is imaged in the real experiment. An example of this live navigation inside a sample is seen in Movie S2 in the Supplementary Materials, where the region of interest to be measured is the interface between two different mineral phases in a piece of volcanic rock. While the sample is continuously rotating, one can easily search for the desired location and accurately position it within the reconstructed field of view shown by the live preview of RECAST3D.

### Capability III: real-time observation of water uptake

Much as the setup alignment and sample positioning are facilitated by the nearly real-time visualisation of reconstructed slices, the main purpose of the presented tool is to allow for the live observation of a dynamical process as it is happening during an experiment. In the case of our yarn sample, this means the observation of the waterfront arrival in the imaged sample region and the subsequent filling of the full yarn’s pore structure with liquid. Once the sample is completely wetted, the measurement can be stopped to avoid the acquisition of unnecessary data. The screencast Movie S3 in the Supplementary Materials shows the whole temporal evolution of the observed sample structure according to the experimental procedure outlined above.

**Figure 5:**
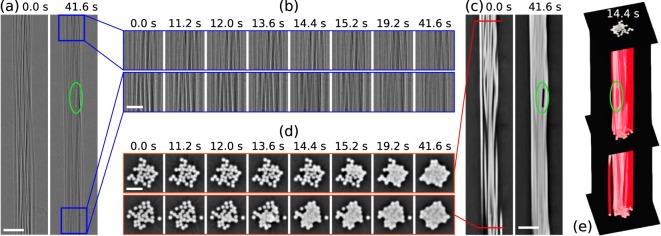
Time series of scans showing the water uptake dynamics of the yarn. (**a**) Radiographic projection images show the whole imaged yarn section at the beginning (0.0 seconds, dry) and the end (41.6 seconds, nearly completely wetted) of the uptake process. Scale bar: 200 *μ*m (**b**) Magnified sections of the radiographic images, indicated by the blue boxes in (**a**), at several time points during the scan at the top (upper line) and bottom (lower line) of the sample. Scale bar: 100 *μ*m (**c**) Vertical centre slice through the phase contrast reconstruction of the yarn sample at the beginning and the end of the scan series. Scale bar: 200 *μ*m (**d**) Horizontal cuts, indicated by the red line in (**c**), through the reconstructed volume at the top and bottom at the same time points shown in (**b**). Scale bar: 100 *μ*m (**e**) Rendering of the reconstructed volume at an intermediate time point during the uptake process, showing one vertical and three axial slices as well as a semi-transparent red isosurface outlining the volume of the combined yarn and water volume. Green outlines: Air bubble remaining in the yarn structure even at the end of the scan series.

Figure 5 shows some representative time points of a data set acquired on an identically prepared sample with 32 fibres instead of 96, imaged under the same experimental conditions. The only difference with respect to the measurement with the live preview was that instead of acquiring one 180 degree scan every two full rotations, a full data set was recorded once per turn, resulting in a scan period of 0.8 seconds instead of the 1.6 seconds used for the scan series visualised on-the-fly with RECAST3D. Panel (a) of Fig. 5 shows flat-field-corrected projection images, or so-called radiographies, of the full extent of the imaged yarn section at the beginning and the end of the water uptake process. Magnified views of small sections at the top and bottom of this imaged region are shown in panel (b) for different time points during the scan series. This is the direct visual feedback tool used so far at most tomographic microscopy beamlines to follow the dynamics of the investigated process. It is essentially impossible to determine when the water front arrives in the two different regions from these projection images as the change in contrast is very small. However, the situation changes dramatically when looking at tomographic slices of the phase-contrast reconstruction. Vertical slices through the centre of the full reconstructed volume are shown for the beginning and the end of the scan series in panel (c). Using axial cuts at the top and bottom of the sample volume, as shown in panel (d), we can readily detect the arrival time point of the leading edge of the water front in the bottom of the imaged region between around 11.2 seconds (still dry) and 12.0 seconds (some pore spaces are filled with water). The same effect is visible in the top slice about 2.5–3 seconds later. The entire time series of flat- and dark field corrected radiographic projection images as well as for the top and bottom reconstructed slices are shown in the Supplementary Materials Movies S4–S6, respectively. A visual rendering of the full 3-dimensional structure (which would not be available in real-time with RECAST3D) for one time point is shown in panel (e) of Fig. 5 with a red semi-transparent isosurface of the water and fibre structure, three axial slices at the bottom, middle, and top of the sample and a vertical slice through the centre of the fibre bundle.

## Discussion

In many cases the information that can be gained from strategically chosen arbitrarily oriented reconstructed slices is a good proxy for the dynamic evolution of the entire sample and is sufficient for adaptive experimental control purposes. By positioning reconstructed slices for instance perpendicular to a front evolution direction, liquid breakthrough can easily be detected. Alternatively, reconstructed slices oriented parallel to it could give a real-time indication of the speed of the propagating front enabling on-the-fly adjustment of the experimental parameters. We believe that if the reconstructed slices are carefully chosen, quasi-3D reconstructions, as the one used in this yarn example, can in many cases provide valuable and representative information for the full 3D structure. The number and type of measurements which could profit from an active automatic feedback will increase with time as new and increasingly optimised tools are being developed by the large and very active image analysis community.

## Outlook: A Route Towards Adaptive Experiment Control

The unprecedented possibility provided by the tools presented here to directly visually follow live dynamic processes as they happen is very valuable for enabling adaptive control of the experiment, for instance to stop the image acquisition and the experiment when the phenomenon of interest is over, so avoiding the storage of a large amount of useless data. Another important application is the case of systems where sudden high-speed events of interest happen only occasionally at essentially unpredictable time points.

Access to a few nearly real-time reconstructed slices through the volume in specifically controlled locations opens up the possibility to go even further and to perform quantitative online analysis on these data. Here we sketch a possible route towards an online feedback mechanism for the presented example of water wicking in yarns, in particular, where we aim to identify the time points of water arrival at the bottom and the top of the imaged sample region, as well as the saturation of the pore volume with water.

**Figure 6:**
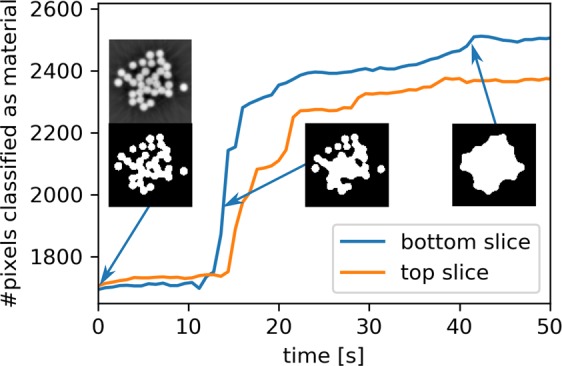
Quantification of the water uptake in the bottom and top slice as a function of time. The reconstructed slices are segmented using a constant threshold and the total number of pixels classified as material is plotted. The insets show the segmented bottom slices at different time points (along with the reconstructed grey-level image for the first time point which was used to automatically determine the threshold for the segmentation).

Judging by the phase-reconstructed slices in Fig. 5(d), identifying the arrival time point of the waterfront should be relatively straightforward. A simple approach relies on the ability to automatically segment the slice data into air and material (in this case, both water and yarn are classified as material). Since the volume of the yarn does not change during the experiment, any change in the amount of detected material can be attributed to water, and a significant rate of change should only be observed starting with the arrival of the water front. Figure 6 plots the total number of pixels per slice classified as material as a function of the scan time. The arrival of the water front is clearly identified as the point when the material fraction suddenly increases. Consistent with the visual inspection, the top slice starts to gain in material about 2–3 seconds after the bottom slice. The small insets show the segmented slices from the bottom of the imaged volume at different time points, using a constant threshold which was determined automatically using the Otsu^24^ method on the dry fibre bundle corresponding to the first time point.

Active feedback to the experiment control in this case could be to start saving data only once the arrival of the water front in the bottom-most slice has been detected and to stop recording data once the water content in both the top and bottom slices has not changed considerably over a given time period. Another option would be, for example, to automatically deliver a staining agent to the water reservoir once the unstained waterfront has reached the imaging region such that the liquid transport in the already wetted yarn can be observed under identical experimental conditions in the same sample as the initial wetting behaviour.

Combining our proposed approach for real-time reconstruction with application-specific postprocessing and visualization operations, the present example can easily be adapted for a broad range of other use cases where the state of the sample must be probed and analysed in real-time to allow for on-the-fly adaptation of experimental parameters.

## Conclusions

The present study demonstrates the feasibility, utility and further potential of the real-time reconstruction of a small number of arbitrarily oriented slices to visually observe the evolution of a sample and to obtain quantitative feedback of the dynamic phenomena occurring during tomographic imaging. The real-time reconstruction has been realised at the TOMCAT beamline at the Swiss Light Source (PSI), and only requires a single workstation for the computations. The chosen approach carefully balances the relative trade-offs between the achievable reconstruction speed, the complexity and cost of the necessary IT infrastructure, and the completeness of the available subset of data during online processing to deliver a powerful quantification and visualisation tool that can be relatively easily integrated into existing data acquisition pipelines with only modest investments into the necessary computing resources.

## Supplementary information

Video S1

Video S2

Video S3

Video S4

Video S5

Video S6

## Data Availability

The software used to generate the results presented this article are RECAST3D (version 658c0e3, 2019-03-08), and SliceRecon (version eb3b4ef, 2019-03-21), both available at https://www.github.com/cicwi as open source projects. The datasets generated and analysed during the current study are available from the corresponding author on reasonable request.
